# Managing Problematic Usage of the Internet and Related Disorders in an Era of Diagnostic Transition: An Updated Review

**DOI:** 10.2174/1745017902117010061

**Published:** 2021-07-14

**Authors:** Bernardo Dell’Osso, Ilaria Di Bernardo, Matteo Vismara, Eleonora Piccoli, Federica Giorgetti, Laura Molteni, Naomi A. Fineberg, Calogero Virzì, Henrietta Bowden-Jones, Roberto Truzoli, Caterina Viganò

**Affiliations:** 1 Department of Psychiatry, University of Milan, Department of Biomedical and Clinical Sciences “Luigi Sacco”, ASST Fatebenefratelli-Sacco, Milan, Italy; 2 Department of Psychiatry, Bipolar Disorders Clinic, Stanford Medical School, Stanford University, Stanford, CA, USA; 3 Aldo Ravelli” Center for Neurotechnology and Brain Therapeutic, University of Milan, 20142 Milan, Italy; 4 Hertfordshire Partnership University NHS Foundation Trust, Rosanne House, Welwyn Garden City, Hertfordshire AL8 6HG, UK; 5 Center for Clinical & Health Research Services, School of Life and Medical Sciences, University of Hertfordshire, Hatfield, UK; 6 School of Clinical Medicine, University of Cambridge, Cambridge, UK; 7 Department of Clinical and Molecular Biomedicine, Psychiatry Unit, University of Catania, Catania, Italy; 8 Central North West London NHS Trust, Division of Brain Science, Imperial College London, London, UK

**Keywords:** Problematic usage of the internet, Management, Internet gaming disorder, Online gambling disorder, Cyberchondria, Cyberpornography addiction, Cyberbullying, Online shopping addiction

## Abstract

**Introduction:**

Problematic Usage of the Internet (PUI) refers to a broad and likely heterogeneous group of Internet-related conditions associated with behavioural disturbances and functional impairment.

**Methods:**

Within PUI several conditions have been reported, including Gaming Disorder, Shopping Addiction, Cyberchondria, Gambling Disorder, Cyberpornography Addiction and Cyberbullying. While increasing reports in the field try to define the epidemiologic and clinical boundaries of these conditions, the rapid and continuous evolution of Internet related behaviours as well as their problematic/pathological expressions are often difficult to diagnose, assess, approach with treatment interventions and follow-up.

**Results:**

In addition, some of the PUI-related conditions show characteristics of addiction to the Internet as a preferential tool to engage in specific behaviours, while some others exclusively manifest on the Internet, making it necessary to find distinct assessment and treatment pathways.

**Conclusion:**

The inclusion of Internet Gaming Disorder in Section III by the DSM-5 and the recognition of Gaming Disorder by the ICD-11 opened the way for a systematic clinical investigation of this and other PUI-related conditions, particularly in terms of preventive and therapeutic strategies. The present article is aimed at offering an updated clinical overview on the main expressions of PUI, focussing on the latest acquisitions in this evolving field.

## 1 INTRODUCTION

Problematic Usage of the Internet (PUI) is an umbrella definition for a growing range of abnormal behaviours that manifest through the use of the Internet [1]. These behaviours share several core features, in particular, the loss of control leads to an increased priority given to online behaviours, resulting in the neglect of other important areas of daily life. Engagement in some forms of PUI (*e.g.*, online gambling) bears the hallmarks of being largely motivated by addiction and being associated with craving, risky or harmful use of the Internet, tolerance and withdrawal symptoms. Behavioural addiction has been defined as repeated behaviour leading to significant distress and consequent functional impairment. This pathological behaviour persists over a significant period of time and can not be limited by the patient [2]. Indeed, the term Internet Addiction, initially proposed in the mid ‘90s [3], remains widely used nowadays to indicate Internet-related psychopathology. In the same period, the pioneering work of several researchers resulted in a set of proposed diagnostic criteria for Internet Addiction that resembled those formulated by the American Psychiatric Association in the Diagnostic and Statistical Manual of Mental Disorders, fourth edition (DSM-IV) for Pathological Gambling [4, 5]. Additional research contributed to elaborate meaningful conceptual differences between addictions on the Internet (those affecting individuals who simply use the Internet as a medium to engage in a specific behaviour that could be conducted offline) versus addictions to the Internet (where individuals are primarily addicted to content solely generated inside the world wide web) [6-9]. At present, it remains relevant to distinguish and clinically characterize different expressions of PUI that represent variants of conditions already recognized as problematic and/or included in the DSM and the International Classification of Diseases (ICD) classification systems.

For instance, this seems to be the case for disorders such as Cyberchondria/Hypochondriasis, Internet Gaming Disorder/ Problematic Videogaming, Cyberpornography Addiction/ Pornography Addiction, Internet Gambling Disorder/Gambling Disorder, Internet Shopping Addiction/Impulsive Compulsive Shopping Disorder, Cyberbullying/Bullying and so on. Nevertheless, between the aforementioned conditions, only Gambling and Gaming Disorder have been currently consi- dered as mental or potential mental disorders. Other forms of PUI, such as digital searching for medical information (*e.g.* Cyberchondria) may share closer similarities with Obsessive Compulsive Disorder and other Obsessive-Compulsive and Related Disorders. These subtypes of PUI appear to be largely motivated by compulsions, defined as irresistible urges to perform stereotyped or rule-bound behaviours aimed at reducing harm or distress [10, 11]. However, different factors overlay and interplay and many forms of PUI showed both addictive and compulsive phenomenology.

Ongoing efforts aim to define the phenomenological and epidemiological boundaries of specific phenotypes of PUI, to document their prevalence rates according to different countries and to identify long-term course and treatment outcomes [12-16]. However, to date, findings related to these issues remain largely inconclusive. While some of the conflicting evidence could be attributed to different methodological approaches and biases, the debate has also been fuelled by experts who feared the potential medicalization and stigmatization of PUI-related conditions and, conversely, by others who stressed the consequences that PUI can have on young people health and lifestyle [1, 2, 17, 18]. Among the firsts, some authors highlighted the risk of misdiagnosing ordinary life behaviours as behavioural addictions. In order to avoid this risk, functional impairment or distress and persistence over time need to be considered as mandatory elements to confirm the diagnosis [2, 19, 20].

In its last edition, the DSM made two important innovations that could pave the way for additional, evidence-based, investigation on PUI. For the first time ever, the DSM acknowledged the existence of behavioural addictions and classified pathological gambling (now Gambling Disorder) alongside substance addictions in the “Substance-related and Addictive Disorders” category (previously considered alongside “Impulse-Control Disorders”) [21]. This decision was based on research that demonstrated clinical, phenotypic, genetic and neurobiological similarities between gambling and substance-use disorders. Gambling Disorder has been therefore classified in this category acknowledging the addictive nature of problematic gambling and providing a possible conceptual framework for the next inclusion of other excessive behavioural patterns, conducted off-line and/or on-line, in the same category as well. The inclusion of Internet Gaming Disorder (IGD) in the DSM-5 Section III as a new condition worthy of further study [22, 23] represented another important and unprecedented change. The inclusion of IGD in the DSM-5 acknowledged for the first time the possible existence of a PUI-related condition as a potential mental disorder and proposed specific diagnostic criteria as the basis for further investigation in the field, including the future definition of other PUI-related conditions. Nevertheless, in the DSM-5 perspective of Internet Gaming Disorder also “non-Internet computerized game” conditions can be included as IGD subtypes. Other non-online repetitive behaviours, named behavioural addictions (*i.e.*, sex, shopping and exercise addictions), are not included in the DSM-5 classification, because of the scarce literature evidence, lack of diagnostic criteria and data on course and outcomes. Most importantly, and building upon these developments, in June 2019 the World Health Organisation (WHO) included Gaming Disorder in the 11th Revision of the International Classification of Diseases (ICD-11) in the substance and behavioural addiction section, though with a slightly different phenomenological approach compared to the DSM-5 perspective and without requiring usage of the Internet as a key component of the disorder.

PUI represents a rapidly evolving field, as shown from the increasing number of investigations focusing on this topic that has almost tripled - in the last five years, compared to the previous five. As a result, clinicians need to be regularly updated about recent advances in this area. The present brief overview of the literature is therefore aimed to present main PUI-related phenotypes, with specific emphasis on pheno- menological, assessment and therapeutic issues, and to summarize current knowledge in this field without providing a systematic review of the literature.

## 2 MATERIALS AND METHODS

A literature search was performed on PubMed and Google Scholar electronic databases to identify recent clinical studies and other literature sources (published in the last 10 years, from January 2010 to January 2020) focusing on the phenomenology and management of PUI-related phenotypes in terms of: 1) characterization; 2) diagnostic and psychometric assessment and 3) therapeutic approaches. In addition, a specific section on prevention strategies was included. A search strategy was performed using the terms: “Internet Gaming Disorder” or “Online Gambling Disorder” or “Cyberchondria” or “Cyberpornography Addiction” or “Cyberbullying”, “Online Shopping Addiction” or “Internet Addiction” or “Problematic Usage of the Internet” and “Assessment” and “Treatment” or “Prevention”. The database search parameters yielded a total of 1158 papers. Among these, 105 studies that only contained anecdotal evidence (*i.e.*, case reports or descriptive paper without measurable psychopathological assessment) or that were not focused on the management of specific PUI subtypes- (*i.e.*, describing generically the negative consequences due to the internet use) were excluded. For the purpose of this study, we did not provide a systematic review of the literature, while an international group of experienced clinicians and researchers in this field selected the most relevant articles focusing on PUI assessment, treatment and prevention. Additionally, reference lists of selected articles were screened. Only articles in English were reviewed. Results are herein presented after grouping PUI-related phenotypes in 6 major conditions: 1) Internet Gaming Disorder, 2) Online Gambling Disorder, 3) Cyber- chondria, 4) Cyberpornography Addiction, 5) Cyberbullying and 6) Online Shopping Addiction.

## 3 RESULTS

### 3.1 Internet Gaming Disorder (IGD)

IGD is a condition characterised by a maladaptive use of the Internet to play video games with negative consequences on individual functioning. Different factors contribute to determining online video games’ reinforcing properties. These include the ease of access a game provides *via* portable or handheld devices, the possibility of engaging in competition with other gamers, the perception of oneself in a manner that is more rewarding and less impacted by real world issues and the specific genres, designs and contents of the games [24-27]. In some countries, high prevalence of IGD has been recorded: nevertheless, epidemiological data on IGD vary, depending on age, country, and screening instruments [28]. Asian countries seem to exhibit a higher prevalence (10-15% among young people in Asian *vs* 1-10% in Western countries). Men, adolescents and young adults are more likely to be problematic gamers [22, 25, 28-32]. Furthermore, IGD often shows comorbidity with anxiety, depression, ADHD or hyperactivity symptoms, social phobia/anxiety, and obsessive-compulsive symptoms [33].

For IGD, the DSM-5 proposed nine different criteria (preoccupation or obsession, withdrawal, tolerance, loss of control, loss of interest, continued overuse, deceiving, escape of negative feelings, functional impairment) and a threshold of at least five criteria lasting for at least of one year to make a diagnosis of IGD. In the DSM-5 perspective, internet access is acknowledged as a relevant feature, even though, as stated in the “Subtypes” paragraph, IGD can involve “non-Internet computerized game”. In addition, biological concepts, such as withdrawal and tolerance, are considered of particular relevance, while less emphasis is given to functional impairment and craving. Time and frequency were not necessarily red flags for PUI, even though adolescents and young adult males, who spend an average of 12 hours per week playing video games, were found to be at higher risk of experiencing problematic and pathological gaming [22, 25, 32]. However, the debate concerning the validity of DSM-5 criteria for IGD is still in progress. Proposed criteria do not probably allow to distinguish an increased but healthy involvement (high engagement) in video games from an excessive and pathological one. This approach may determine several false positives and an over pathologization of normal behaviours, failing to take into account that repeated and high engagement is not problematic *per se*. The engagement construct underlines a harmonious passion for video games, without negative consequences and functional impairment. Similarly to the present conceptualization of IGD, the diagnosis of gaming disorder in the upcoming ICD-11 classification should focus on the impairing nature of gaming from a functional point of view [2, 34-36].

According to ICD-11, a patient must exhibit 3 core symptoms to be diagnosed with Gaming Disorder (GD): impaired control over gaming, increasing priority given to gaming, continuation or escalation of gaming despite the occurrence of negative consequences. In the ICD-11 approach, both online and offline game accesses are acknowledged, with significant emphasis on functional impairment and, therefore, on more severe expressions of PUI [37].

The first review focusing on the quality of IGD psychometrics tools has been published in 2013, just before the launch of the DSM-5. The authors identified withdrawal, loss of control and conflict as the defining features of IGD [38]. Since then, the situation has not seen a substantial evolution. More recently, an article from a Spanish group identified seven principal assessment instruments, developed after DSM-5 introduction of IGD diagnosis: Internet Gaming Disorder Test (IGD-20); Internet Gaming Disorder Scale – Short Form (IGDS9-SF); Problematic Online Gaming Questionnaire (POGQ); Problematic Online Gaming Questionnaire – Short Form (POGQ-SF); Video Game Addiction Test (VAT); Clinical Video Game Addiction Test (C-VAT 2.0); and Internet Gaming Disorder Scale (IGDS), (Table **1**) [39]. Overall, the IGDS9-SF was found to be the most widely used assessment tool worldwide, with numerous validations and translations into different languages. The IGDS9-SF [40] is a brief instrument consisting of nine items covering the nine DSM-5 diagnostic criteria. This tool aims to measure the severity of IGD and its pathological consequences, assessing online and offline gaming activities during the previous year. The IGD-20 needs to be mentioned for its ability to examine different gaming profiles and represents a valid and accurate tool to assess IGD, incorporating DSM-5 diagnostic criteria and reflecting the six dimensions of the *Griffiths’ addiction model* (2005): salience, mood modification, tolerance, withdrawal, conflict, and relapse [39, 41]. The POGQ consists of a 26 item questionnaire organized in six dimensions: preoccupation, overuse, immersion, social isolation, interpersonal conflicts, and withdrawal symptoms [42]. POGQ-SF comprises 12 items reflecting the six POGQ dimensions [43]. The VAT is a direct adaptation of the items of the Compulsive Internet Use Scale (CIUS), focusing specifically on video game playing, elaborated before the appearance of the DSM-5. C-VAT 2.0 is an adaptation of the C-VAT to the DSM-5 criteria. C-VAT 2.0 is composed of 11 items (Yes/No) about IGD symptoms in the previous 12 months (nine of which covering the nine DSM-5 criteria for IGD) [44]. IGDS is a 27-item German-language tool in which each DSM criterion is assessed through three items [45].

In relation to therapeutic approaches to IGD, several studies investigated the potential utility of different psychopharmacological agents, but, currently, no medication has been approved for this condition [24, 46]. As far as antidepressants are concerned, the administration of Bupropion has shown a reduction of average time spent online and craving dimension in open-label [47, 48] and randomized controlled studies [49]. Two open trials comparing the effectiveness of Bupropion and Escitalopram showed that both antidepressants were effective in reducing IGD symptoms, though Bupropion was more effective in improving attention and in impulsivity decrease [50, 51]. In a case report, Citalopram was combined with an antipsychotic in a young patient, showing an improved control over Internet use and decreased addiction [52]. Indeed, further studies are needed to investigate the efficacy of Selective Serotonin Reuptake Inhibitors (SSRIs) in the treatment of IGD [53]. Due to the high comorbidity with ADHD, the use of Methylphenidate and Atomoxetine has been investigated in a recent 3-month prospective trial, showing a significant improvement of ADHD and IGD symptoms [54].

Nevertheless, further studies are needed in this field, since available data show several limitations concerning the lack of standardized assessment and definitions of IGD, determining various difficulties in comparing treatment outcomes across studies. Additional limitations include small samples and lack of control groups, randomization, blinding, and key details like sample characterization [24, 45, 55].

With respect to psychological treatment approaches for IGD, several interventions have been assessed: Cognitive-Behavioural Therapy (CBT), motivational interviewing, family therapy, educational courses focused on speaking and writing, basic counselling, solution focusing-therapy, reality training and a combination of them [46, 56-61]. Among them, CBT has been the most extensively studied [55, 58, 62] thanks to four randomized controlled trials [59, 63-65]. CBT demonstrated the most robust evidence in terms of efficacy in reducing weekly gaming hours and IGD symptoms, showing the largest evidence base versus other therapies [57], [66-68]. A recent review by King and colleagues (2017) pointed out a stronger consensus on the benefits of CBT compared to other therapeutic approaches, particularly in comparison to pharmacological treatment [46]. More recently, another review showed that CBT, which is often considered the first-line therapy for IGD, can improve both primary IGD symptoms and comorbid depression. However, treatment improvements usually manifest in the short-term and their effect in reducing time spent gaming is unclear [69]. Concerning brain stimulation techniques, a 4-week Transcranial Direct Current Stimulation protocol was found to be useful for reducing gaming online and enhancing self-control, likely by improving interhemispheric balance of glucose metabolism in the Dorsolateral Prefrontal Cortex [70].

### 3.2 Online Gambling Disorder (OGD)

OGD refers to placing money at stake in the hope of a large pay-out, with gambling outcomes being only partially or not at all skill dependent [71]. Compared to traditional gambling, the use of the Internet allowed new types of online gambling, along with traditional ones (*e.g.* poker, casinos, sports betting), making OGD one of the most popular and lucrative businesses on the Internet. Internet gambling represents a fundamental shift in the way consumers engage in gambling, and several preoccupations have been expressed by various stakeholders about these changes. Specific concerns and disadvantages reported by Internet gamblers highlight the easy and convenient possibility to spend money online and the high accessibility to online gambling, likely increasing the risk for OGD particularly among technology-savvy youths [72, 73]. Consequently, the Internet represents a facilitating factor in the development of pathological gambling in vulnerable individuals, since it favours access to gambling possibilities [74]. This feature can perhaps explain why the prevalence of problematic gambling appears to be at a higher level among online gamblers than non-online gamblers [75].

On the other hand, an advantage of online gambling is that “self-exclusion” from gambling websites may be more effective than from offline gambling for those who wish to stop gambling. Indeed, in some countries (*i.e.*, in the UK), specific websites concerning self-exclusion are available for the patients [76, 77].

Currently, literature data are not conclusive in confirming OGD as being more harmful or more problematic than offline gambling [78]. In terms of sociodemographic features, online pathological gamblers seem to be much younger than offline gamblers (*i.e.*, 30 years old compared to 40 years old on average) [78]. Overall, in adolescents online gamblers, compared with offline gamblers, a stronger link between OGD severity, problematic alcohol use and poor academic performance was found [79].

OGD can be assessed with a 3-item screening test, the National Opinion Research Centre DSM Screen for Gambling Problems (NODS-CLiP) [80, 81]. According to the NORC (National Organization for Research at the University of Chicago) DSM-IV Diagnostic Screen for Gambling Disorders, the NODS-CLiP investigates the three main areas of malfunctioning: loss of control, lying and preoccupation. It was demonstrated that there are high rates of specificity and sensitivity in diagnosing Gambling Disorder when patients endorse one or more of these items [81]. When the diagnosis is certain, however, the most used tool is Problem Gambling Severity Index (PGSI), also available for OGD, a 9-item questionnaire validated in 2009 that measures gambling severity in the last 12 months, with a cut-off score of 8 indicating patients as “problem gamblers” [82, 83].

Preliminary data showing an earlier onset of pathological gambling in online gamblers compared with offline ones suggests that treatment may require more effort for online subtypes, including online interventions and dedicated public youth departments, as well as strategic partnerships with schools [78]. Currently, OGD treatment is comparable to Gambling Disorder one, without specific measures for online behaviours. No pharmacological treatments have received official indication for gambling disorder, although placebo-controlled trials suggest that medications, as opioid receptor antagonists, may be helpful. Naltrexone is one of the most investigated molecules for gambling disorder [84-88]. In one of the first Randomized Controlled Trials (RCTs) in the field, considering subjects reporting moderate or strong urges to gamble, naltrexone performed better than placebo in reducing gambling symptoms [86]. In a follow-up study, in which patients were randomized in a double-blind trial and assigned to one of the three different daily doses of naltrexone (50 mg, 100 mg or 150 mg) or to placebo, Naltrexone exhibited a greater reduction in gambling urges than placebo, with no significant differences between the different drug doses [85].

As far as Nalmefene is concerned, limited evidence has been published. In a double-blind, placebo-controlled trial design, patients prescribed Nalmefene showed greater improvements in problem-gambling severity compared to placebo [89]. Another multicentre trial reported less positive results for Nalmefene. However, in the post-hoc analyses, the group on Nalmefene had a greater reduction in problematic gambling severity than the placebo group [90].

Specific evidence on the utility of pharmacological therapy in OGD is scarce. Of note, a 12-week open-label study on 14 patients treated with Bupropion up to 300 mg/day highlighted a significant improvement of clinical symptoms [47]. In terms of psychotherapeutic interventions, a recent study demonstrated that Internet based CBT could improve access to treatment among non-help -seeking online gamblers [91]. Concerning psychological treatment, CBT has been reported to be the most commonly used intervention in and offline pathological gambling [92]. A recent review has specifically recommended 6 to 8 sessions of CBT to integrate motivational interventions. For subjects with less severe gambling, minimal interventions involving feedback related to one’s gambling may also be effective [93].

In conclusion, considering OGD treatment opportunities and according to the therapy of Gambling Disorder, the combined approach (psychotherapy and pharmacotherapy) could be more engaging than pharmacotherapy alone and provide individuals relevant and useful skills that could lead to sustained treatment improvements [94].

### 3.3 Cyberchondria (CYB)

CYB has been defined as an excessive or repeated online searching for health-related information, which is driven by a need to alleviate distress/anxiety about health, and ultimately results in the worsening of such symptoms and behaviours [10, 11]. CYB resembles an urge driven compulsive/repetitive behaviour and manifests a time-consuming nature. Very limited descriptions of CYB in clinical samples are available, with all studies investigating this behaviour in the general population/university students, mainly recruited through online surveys. A consensus on CYB definition is still lacking, but the link between CYB and other psychiatric disorders has been established in the literature. CYB appears to be a transdiagnostic multidimensional construct with a strong correlation with PUI, health anxiety/hypochondriasis and Obsessive-Compulsive and Related Disorders [95-98].

A 33-items self-reported questionnaire, the “Cyberchondria Severity Scale”, is a continuous severity assessment tool to measure CYB that explores 5 domains: compulsion, distress, excessiveness, reassurance, and mistrust of medical professionals [99]. A shorter 12-items (4 domains) questionnaire has been validated and is potentially more suitable for clinical and research application.

No studies have investigated the efficacy of any therapeutic approach in CYB. Psychoeducation and CBT have been proposed. Considering the strong link between CYB and health anxiety/hypochondriasis, a reasonable pharmacological intervention might be the use of medications that proved to be efficacious in hypochondriasis [26, 100]. Therapeutic approaches should be extended to a public health level in order to improve the way health related information is presented and accessed online.

### 3.4 Cyberpornografy Addiction (CPA)

When the act of using Internet to view or interact with pornographic material [101] becomes excessively time consuming, distressing and difficult to resist, it indicates the potential presence of a pathological behaviour named Cyberpornograpy addiction (CPA). Around 12% of cyberpornography users tend to become compulsive, showing comorbid, recurring and uncontrollable sexual concerns that favour adverse consequences [102, 103]. cyberpornography addicts were found to spend on average 110 minutes per day watching pornography and reported high levels of compulsivity and emotional distress, with sexual avoidance and low sexual gratification and impairment in several areas of functioning [103-105].

The Cyber Pornography Use Inventory (CPUI), a 31-item questionnaire, is the most widely used instrument to assess the three dimensions of pornography use: compulsivity, intensity of efforts to access pornography and emotional distress associated with pornography use [103, 106]. Another tool is the Internet Sex Screening Test (ISST) item, a 25 true-false item test that identifies low risk (1-8), at risk (9-18) and high risk (> 19) abnormal Internet sex behaviours [107, 108].

To date, evidence on the efficacy of psycho- pharmacological treatment for CPA is very limited: only scattered case reports and one review addressed this topic [105, 109-112]. Naltrexone was used in three patients, either as monotherapy or adjunct therapy, with doses up to 150 mg/day, with an improvement in functioning and a reduction in the time spent online [105, 109, 110].

Paroxetine was administered along with CBT in three male patients, showing mixed results: although a short-term reduction of anxiety and pornography use was observed, new compulsive sexual behaviours emerged later during the observation [111].

As far as psychotherapy is concerned, CBT, Couple Therapy, Structural therapy, Online Psycho-educational program and Acceptance and Commitment therapy showed a positive effect in reducing symptoms severity [112].

### 3.5 Cyberbullying

The term “cyberbullying” was coined to describe the use of digital technology to seek to harm, intimidate, or coerce other people online [113-115]. This behavior has reached the attention of scientific study in order to increase the knowledge about its causes and effects on the bully and on the victims. A strong association between cyberbullying and mental health problems (*i.e.*, social anxiety, depressive disorders, substance abuse and suicide) has been reported [116].

Despite the severity of this condition, limited assessment instruments have been developed so far [117]. The Cyberbullying Questionnaire is an 88-item test whose purpose is to analyse the incidence of cyberbullying in and out of schools [115]. Two recent reports proposed novel assessment instruments: the Cyberbullying Test with confirmed reliability and validity and the Cybervictimization Emotional Impact Scale which assess the emotions experienced by victims and bullies [117, 118].

So far, validated interventions for cyberbullying in the healthcare setting do not exist. In a recent systematic review, several different programs were discussed and individual intervention components (like digital citizenship, coping skills, education on cyberbullying, communication and social skills and empathy) were identified and their inclusion into clinical practice guidelines could reduce both cyberbullying and cybervictimization [119].

In a recent study, the effectiveness of “Prev@cib program”, based on three theoretical frameworks including ecological model, empowerment theory and personal and social responsibility model, was evaluated. Results showed a significant decrease in cyberbullying and cybervictimization [120].

### 3.6 Online Shopping Addiction (OSA)

OSA may be classified in the category of specific addiction *on* the Internet [1, 121-125] and it has been defined as a tendency to excessively and compulsively shop online that results in economic, social and emotional negative consequences [126].

E-commerce specific characteristics, such as products immediate availability, anonymity, easy accessibility and affordability provide a range of potential addictive features that reinforce OSA development [127-129]. Indeed, online shopping could be considered more addictive than shopping in the real world. In terms of individual characteristics, deficient self-regulation has been identified as the most important predictor for OSA [130, 131]. Prevalence data are not conclusive: a study found that 33.6% of compulsive shoppers manifested OSA [132]. In terms of assessment, the OSA scale, an 18-item questionnaire, has been developed to empirically measure OSA. This tool is based on previous research on a widely accepted six-factor component model (salience, mood modification, tolerance, withdrawal, conflict and relapse) [133]. The OSA scale scores predicted the self-perceived OSA to a relative high degree [126].

As far as treatment is concerned, no studies specifically focused on OSA. Yet, a feasible approach might be the adoption of interventions approved for comparable forms of behavioural addiction, such as Buying Disorder. In this respect, the use of antidepressants, mood stabilizers, opioid antagonists and CBT might be valuable options [134, 135].

In respect to psychotherapeutic interventions, a recent review found that the CBT was effective for the treatment of compulsive buying disorder, making this approach potentially applicable for the treatment of OSA as well [136].

### 3.7 PUI Prevention

Studies on prevention strategies for PUI are at a preliminary level: scarce RCTs were identified and frequently PUI was not the main focus of the investigation [137-141]. Researchers suggest that prevention programs for Internet-related disorders should mainly focus on children and adolescents [142, 143], using psycho-educational interventions, supporting resilience and adaptive coping strategies [144]. Considering an evolutive approach, several East Asian countries made a strong investment on primary prevention in schools [137, 143, 145-147]. Primary prevention should be complemented with an ecological perspective that includes the individual’s and familiar environment, social networks community and public policies [148]. In clinical settings, an evaluation of mental and psychological status might be useful to identify the presence of factors that might promote a maladaptive use of the Internet (*i.e.*, personality traits, physiological characteristics, Internet use pattern and sociodemographic and clinical variables) [149-157].

With respect to secondary prevention, a valid strategy lies on the implementation of screening procedures in schools in order to measure the frequency of PUI [138, 144, 158-160]. However, it is important to state that interventions should be tailored on each specific phenotype of PUI. For instance, a recent review suggested that cyberbullying could be prevented by providing information about bullying to children, parents and school personnel through websites and other online resources and by implementing psychoeducational programs promoting empathy, perspective taking, communication skills, problem solving, and friendship skills [161]. Considering IGD, a useful strategy is *“parenting”* that consists of preventive approaches aimed at promoting a responsible use of the Internet. In particular, parents and responsible adults should put limitations on screen time for children and stimulate a more beneficial usage of this tool [32].

Considering tertiary prevention, some likely useful approaches include: self-help groups, psychoeducational interventions and social-skills rehabilitation [162]. Preliminary data suggest that mindfulness techniques may reduce craving and emotional dyscontrol [163, 164]

## 4 DISCUSSION

Management of PUI-related disorders represents an important challenge involving patients, families, psychiatrists, psychologists and social workers, with multiple repercussions on the whole society. A larger consensus on diagnosis and characterization of main PUI-related phenotypes, assessment, treatment and prevention is required in order to carry out evidence-based clinical interventions.

Previously, abnormal Internet-related behaviours mainly concerned Asian countries, but it is now established that PUI represents a global emerging public health issue, with relevant societal costs [13, 15].

In spite of the increasing reports on the cost and burden of PUI, specific clinical services for early detection and management are at this point limited and present only in some countries, and professional guidance and help are often limited or unavailable. Ultimately, inadequate resources and poor knowledge in the field of PUI contribute to its underdiagnosis and undertreatment. Lack of awareness, in turn, results in poor early intervention strategies, potentially contributing to the onset of secondary comorbid disorders and significant functional impairment (*e.g.*, lower school-work performance). Conditions like insomnia, anxiety, depression, dysphoria, social phobia and social withdrawal (*i.e.*, Hikikomori: a Japanese term meaning “being confined”, as an abnormal avoidance of social contact), and health risks, especially in the youth (*i.e.*, a diet comprised of junk food, lack of exercise, obesity, postural pain syndromes in cervical or lumbar spine, repetitive stress injury) represent some of the most frequently observed correlates of untreated PUI. Achieving consensus on the definition, clinical course and assessment of different phenotypes of PUI, could be expected to reduce the burden of disease and help to identify the critical factors that are relevant for successful preventive interventions. In order to reduce the damaging consequences of PUI, the development of a valid and structured prevention program involving public health awareness and family involvement that extends to involve schools and local communities is needed.

Even though some progress has been made in recent years, in regard to PUI therapeutic strategies, it is still difficult to draw conclusions, due to methodological limitations and lack of adequate and homogeneous studies on specific therapeutic interventions. Overall, there are two overarching approaches to the treatment of behavioural addictions: the first one is to administer symptomatic therapy according to the clinical presentation and/or theoretical model of the disease, based on the pharmacological interventions approved for other forms of addiction. In this respect, it is useful to clarify whether the symptoms can belong to the obsessive-compulsive, impulsive or addictive spectrum. The second approach consists of intervening on comorbid mental disorders. In this regard, it is still unclear whether the chosen therapy merely addresses the potentially comorbid psychiatric disorder or is effective in treating PUI *per se* [165, 166]. Theoretical models on the pathogenesis of other substance and behavioural addictions provide a valuable rationale for the use of specific pharmacological classes, such as glutamatergic drugs (*e.g.*, Memantine, Riluzole, metabotropic glutamate receptor agonists), mood stabilizers (antiepileptic drugs, lithium), opioid receptor antagonists (Naltrexone) and alpha-2 adrenergic receptor agonists [165, 166]. Regarding psychotherapeutic approaches, a review conducted by Liu and colleagues in 2011, which did not focus on specific PUI subtypes, indicated CBT combined with family therapy or group therapy as possibly effective for the treatment of Internet Addiction [167].

In a more recent meta-analysis (2017), counselling programs, cognitive behavioural therapies, and sport interventions were found to show a significant effect on Internet Addiction and related psychopathological symptoms [168]. Further studies need to be conducted in order to prove the effectiveness of these therapeutic tools, across various phenotypes of PUI. Unfortunately, at the present time, methodological problems limit the confidence in the conclusions that can be drawn from any of the aforementioned reports on therapeutic approaches. As recently pointed out by participants to the 4-year Cooperation in Science and Technology (COST) action “Manifesto for a European research network into Problematic Usage of the Internet”, investigation priorities for the next years in the field of PUI in order to improve the understanding of its different expressions include the needs to: achieve a consensus-driven conceptualisation of PUI; elaborate reliable assessment tools; establish the impacts on health and daily life; describe the clinical courses; encourage early interventions to remove risk factors; define the role of genetics and personality features; clarify the impact of social factors; validate effective interventions, both to prevent and to treat PUI and identify biomarkers as well as to improve early diagnosis [1].

While, to date, the scientific community and the priorities of mental health policy makers have largely overlooked the field of behavioural addictions and PUI, the time is now right for cultural change based on the acquisition of reliable epidemiological, clinical and social evidence. Public partnership with researchers, clinicians and health policy makers represent a key ingredient in the successful accomplishment of this task.

## CONCLUSION

In conclusion, the present report was conceived as a non-systematic review of the literature that aims to describe different expressions of PUI with distinct prevalence rates, assessment tools and clinical characteristics. However, despite a growing body of evidence on the impact of Internet-related psychopathology on a personal and societal level, PUI needs additional research, in particular considering the widespread precocious exposure to digital technology, whose potential consequences are almost unknown. Certainly, the decision to acknowledge the existence of “behavioural addictions” operated by DSM-5 and ICD-11 through the inclusion of gambling and gaming disorders within the chapters of addictive disorders will provide new opportunities to implement an evidence-based approach for the investigation of PUI: a series of conditions with urgent need of rigorous field studies, particularly in the areas of prevention and therapeutic interventions.

The present report should be considered in light of the following limitations. First, the article stands as an overview presenting state of the art in the field of PUI and related conditions, without the aim to conduct a systematic review of the literature. Second, only Pubmed and Google Scholar electronic databases were considered as sources. Third, due to space limitation, it was not possible to discuss in detail every cited study. Last but not least, the present article was intended to be as inclusive as possible, but in light of the aforementioned limitations, we had to select only some of the most representative PUI related conditions. Therefore, other PUI expressions, including Cyberstalking, Cyberhoarding, and problematic forms of general web-surfing and mail checking, which also share a time-consuming and disabling use of the Internet, were not considered. Finally, some of the covered phenotypes exhibit an evident phenomenological heterogeneity (*i.e.*, Cyberbullying, differently from other conditions, is not characterised by compulsiveness) which should be further characterized.

## DISCLOSURE

Prof. Dell'Osso has received Grant/Research Support from LivaNova, Inc., Angelini and Lundbeck and Lecture Honoraria from Angelini, FB Health and Lundbeck.

Prof. Naomi A. Fineberg declares that in the past 3 years she has held research or networking grants from the ECNP, UK NIHR, EU H2020, MRC, University of Hertfordshire; she has accepted travel and/or hospitality expenses from the BAP, ECNP, RCPsych, CINP, International Forum of Mood and Anxiety Disorders, World Psychiatric Association, Indian Association for Biological Psychiatry, Sun; she has received payment from Taylor and Francis and Elsevier for editorial duties. In the past 3 years, she has accepted a paid speaking engagement in a webinar sponsored by Abbott. Previously, she has accepted paid speaking engagements in various industry supported symposia and has recruited patients for various industry-sponsored studies in the field of OCD treatment. She leads an NHS treatment service for OCD. She holds Board membership for various registered charities linked to OCD. She gives expert advice on psychopharmacology to the UK MHRA.

## Figures and Tables

**Table 1:** PUI-related disorders: Assessment and treatment approaches.

**PUI Phenotype**	**Assessment**	**Pharmacological Treatment**	**Non Pharmacological Treatment**
**INTERNET GAMING DISORDER (IGD)**	IGD-20 (examine different gamer profiles) IGDS9-SF (the most used) POGQ POGQ-SF VAT C-VAT 2.0 IGDS	**2 reviews, 1 case report,** ** 6 clinical studies** Escitalopram up to 20 mg/die *(Song *et al.*, 2016; Nam *et al.*, 2017)* Bupropion up to 300 mg/die* (Han *et al.*, 2011; Han and Renshaw., 2012; Song *et al.*, 2016; Nam *et al.*, 2017; Bae *et al.*, 2018)* Citalopram up to 40 mg/die + Quetiapine up to 200 mg/die *(Atmaca *et al.*, 2007)* Methylphenidate up to 40 mg/die and Atomoxetine up to 60 mg/die *(Park *et al.*, 2016*)	**2 reviews, 3 clinical studies** Psychological approach: CBT (best evidence) motivational interviewing reality training combination of psychological and/or counseling therapies *(King *et al.*, 2017; Zajac and M.K, 2017)* Specialized psychotherapy: PIPATIC program *(Torres-Rodriguèz and Carbonell., 2017; Torres-Rodriguèz *et al.*, 2017*) brain stimulation: tDCS on DLPFC (4 weeks) *(Sang *et al.*, 2018)*
**ONLINE GAMBLING DISORDER (OGD)**	NODS-CLiP (3-item screening test) PGSI (9-item test to assess severity)	**1 clinical study** Bupropion up to 300 mg/die *(Bae *et al.*, 2018)*	**1 clinical study** internet based CBT without guidance should improve access to treatment among non help-seeking online gamblers *(Luquiens *et al.*, 2016)*
**CYBERCHONDRIA (CYB)**	CSS (33-items, 5 domains) CSS-SF (12-items, 4 domains)	No studies SSRIs have been proposed	No studies CBT has been proposed
**CYBERPORNOGRAFY ADDICTION ** **(CYA)**	ISST (25 true-false item screening test) CPUI (31-items to assess severity)	**1 review, 2 case series, 2 case reports** Naltrexone as a monotherapy or as an adjunct therapy and with doses up to 150 mg/d *(Bostwick *et al.*, 2008; Kraus *et al.*, 2015; Capurso, 2017)* Paroxetine 20 mg/die and CBT *(Gola and Potenza, 2016)*	**1 review** CBT, Adlerian Counselling, Structural therapy, Couple Therapy, Structural therapy, Online Psycho-educational program, Acceptance and Commitment therapy, Group therapy. *(Sniewski *et al.*, 2018)*
**CYBERBULLYING**	CQ (88-item ) CT (equipped with psychometric properties) CVEIS (regarding victim and bullies emotions)	No studies	**1 review, 2 clinical studies** The most frequently proposed interventions included components like education on cyberbullying for the adolescent, coping skills, empathy training, communication and social skills, and digital citizenship *(Hutson *et al.*, 2018)* Prev@cib program *(Ortega-Baròn *et al.*, 2019)* RPC (Relazioni Per Crescere) teacher-based program *(Guarini *et al.*, 2019)*
**ONLINE SHOPPING ADDICTION (OSA)**	OSASS (predicted the self-perceived online shopping addiction )	No studies specifically focused on OSA, yet a feasible approach might be the adoption of interventions approved for comparable forms of behavioural addiction	No studies specifically focused on OSA, yet a feasible approach might be the adoption of interventions approved for comparable forms of behavioural addiction

IGD-20:Internet Gaming Disorder Test (IGD-20); IGDS9-SF: Internet Gaming Disorder Scale – Short Form; POGQ: Problematic Online Gaming Questionnaire;

POGQ-SF:Problematic Online Gaming Questionnaire – Short Form; VAT: Video Game Addiction Test; C-VAT 2.0 Clinical Video Game Addiction Test

IGDS: Internet Gaming Disorder Scale;

NODS-CLiP National Opinion Research Centre Diagnostic and Statistical Manual of Mental Disorders Screen for Gambling Problems

CBT: cognitive-behavioral therapy; MI: motivational interviewing

RT: reality training; t-DCS: Transcranial Direct Current Stimulation; DLPFC: Dorsolateral Prefrontal Cortex (DLPFC)

PGSI: Gambling severity is the Problem Gambling Severity Index; CSS:Cyberchondria Severity Scale; CSS-SF:Cyberchondria Severity Scale Short Form

CPUI: Cyber Pornography Use Inventory; ISST: Internet Sex Screening Test (ISST; CQ: Cyberbullying Questionnaire

CT:Cyberbullying Test; CVEIS: Cybervictimization Emotional Impact Scale; OSASS: Online Shopping Addiction Scale Scores
